# Berberine Attenuates Intestinal Mucosal Barrier Dysfunction in Type 2 Diabetic Rats

**DOI:** 10.3389/fphar.2017.00042

**Published:** 2017-02-03

**Authors:** Jing Gong, Meilin Hu, Zhaoyi Huang, Ke Fang, Dingkun Wang, Qingjie Chen, Jingbin Li, Desen Yang, Xin Zou, Lijun Xu, Kaifu Wang, Hui Dong, Fuer Lu

**Affiliations:** ^1^Institute of Integrated Traditional Chinese and Western Medicine, Tongji Hospital, Tongji Medical College, Huazhong University of Science and TechnologyWuhan, China; ^2^Department of Integrated Traditional Chinese and Western Medicine, Tongji Hospital, Tongji Medical College, Huazhong University of Science and TechnologyWuhan, China; ^3^Department of Biochemistry and Molecular Biology, Tongji Medical College, Huazhong University of Science and TechnologyWuhan, China; ^4^Department of Pharmacy, Hubei University of Traditional Chinese MedicineWuhan, China

**Keywords:** berberine, diabetes, intestinal mucosal barrier, gut immune system, gut-derived hormones

## Abstract

**Background:** Intestinal mucosal barrier dysfunction plays an important role in the development of diabetes mellitus (DM). Berberine (BBR), a kind of isoquinoline alkaloid, is widely known to be effective for both DM and diarrhea. Here, we explored whether the anti-diabetic effect of BBR was related to the intestine mucosal barrier.

**Methods and Results:** The rat model of T2DM was established by high glucose and fat diet feeding and intravenous injection of streptozocin. Then, those diabetic rats were treated with BBR at different concentrations for 9 weeks. The results showed, in addition to hyperglycemia and hyperlipidemia, diabetic rats were also characterized by proinflammatory intestinal changes, altered gut-derived hormones, and 2.77-fold increase in intestinal permeability. However, the treatment with BBR significantly reversed the above changes in diabetic rats, presenting as the improvement of the high glucose and triglyceride levels, the relief of the inflammatory changes of intestinal immune system, and the attenuation of the intestinal barrier damage. BBR treatment at a high concentration also decreased the intestinal permeability by 27.5% in diabetic rats. Furthermore, BBR regulated the expressions of the molecules involved in TLR4/MyD88/NF-κB signaling pathways in intestinal tissue of diabetic rats.

**Conclusion:** The hypoglycemic effects of BBR might be related to the improvement in gut-derived hormones and the attenuation of intestinal mucosal mechanic and immune barrier damages.

## Introduction

The number of diabetics underwent a four-fold change over the past 35 years, and worldwide incidence of diabetes mellitus (DM) was about 9% in 2014 (Lancet, 2016a). In China, the incidence was 11.6%, and the rate of prediabetes was 50.1% among adults. However, only 25.8% of diabetics accepted hypoglycemic treatment, and only 39.7% population of the treated diabetic patients achieved eligible glucose control (Xu et al., 2013). In 2012, it was reported that poor blood glucose control led to the deaths of 370 million diabetics (Lancet, 2016b). High mortality rate of diabetes brings great health burden to the world populations, arousing researchers to seek out new therapies of diabetes based on new insight into the pathogenesis. Among these, the role of intestine has attracted great concerns.

The intestinal immune system is the first defense line for the contact with dietary antigens. Intestinal changes caused by intestinal infection, oral milk or gluten protein antigens, and varied intestinal flora has been confirmed involved in the pathogenesis of DM (Luck et al., 2015). Prior to the occurrence of diabetes, the intestinal morphology and gut immune system have changed, and intestinal permeability increases (Graham et al., 2004). On the other hand, mesenteric lymphocytes transplantation of non-obese diabetic mice could transfer diabetes to the recipient mice, which indicates DM-caused T cells exist in the gut immune tissue (Antvorskov et al., 2014). As is well known, T2DM is a chronic inflammatory disease; many factors are associated with the onset of T2DM including intestinal flora disturbance, immune tolerance deficiency, intestinal barrier damage, pattern recognition receptor (PPRs) expressions changes in intestinal epithelial and immune cells and gut hormone change (Winer et al., 2016). With intestinal barrier defect, LPS and other intestinal bacteria products filter into the circulation and result in local and systemic chronic inflammation, which makes islet beta cell dysfunction and insulin resistance (Burcelin, 2012). However, the role of gut immune cells and inflammation in the pathogenesis of DM are still not fully explored.

Berberine (BBR, [C20H18NO4]+), a kind of isoquinoline alkaloid, could play hypoglycemic and hypolipidemic roles in clinical and experimental studies (Dong et al., 2012, 2013). Some studies illuminated diversified action mechanisms of BBR for treating diabetes. For example, BBR could increase the glucose uptake and consumption in adipose and muscle cells, elevate insulin receptor expressions of liver and muscle, and enhance liver low density lipoprotein receptor expressions to decrease serum cholesterol and glucose (Pirillo and Catapano, 2015). Moreover, Th1 and Th17 cells differentiation and macrophage trafficking could be blocked by BBR, thereby proinflammatory cytokine secretions are reduced (Pirillo and Catapano, 2015). However, BBR is extensively known as a medicine for the treatment of diarrhea in China. In the gut, BBR could stimulate the secretions of GLP-1, depress the activity of α-glucosidase and reduce the intestinal absorption of glucose (Leng et al., 2004; Turner et al., 2008; Zhao et al., 2012).

Taken the low oral bioavailability conundrum of BBR as well as its effect on diarrhea into account (Pirillo and Catapano, 2015), we hypothesized that BBR might prevent the development of diabetes through its action on the intestines. Metformin have also been shown to alter the intestinal flora, modulate gut hormones and maintain the intestinal barrier integrity (Shin et al., 2014; DeFronzo et al., 2016; Mardinoglu et al., 2016). Therefore, we conducted a study to testify whether the hypoglycemic effects of BBR as well as metformin were via preventing intestinal mucosal barrier damage and stimulating gut hormones release.

## Materials and Methods

### Animals, Grouping and Modeling

Male Wistar rats with weight between 160 and 180 g were purchased from Hua Fukang Co., Ltd and bred in SPF circumstances in the experimental animal center of Huazhong University of Science and Technology. After acclimatization for 2 weeks, the rats were randomly divided into normal group (NO group) and HGFD group (HGFD group). The rats in NO group was fed with standard laboratory rat chow (the formula contains 35% flour, 20% corn meal, 20% soy meal, 15.5% bran, 5% fish meal, 1% dusty yeast, 0.5% bean oil, 2.5% bone meal, and 0.5% salt) while the diet in HGFD group contained 67.5% standard laboratory rat chow, 12% lard, 2% cholesterol, 0.5% bile salts and 20% sugar. The dietary regimens of the rats in different groups were unchanged until the end of the experiment. One month later, intravenous injection of small dose streptozotocin (STZ, 24mg/kg) was conducted in the rats of HGFD groups (Duca et al., 2015). Then the normal 95% confidence intervals at various time points in OGTT were achieved according to the blood glucose of rats in NO group. If the blood glucose was greater than 20% of the upper limit of confidence interval at any time point, the rats were chosen for further studies as successful models of diabetic rats. Diabetic rats were further randomly divided into model group (MO group) and treatment groups, with 12 rats in each group. All experiments were approved by the animal ethics committee of Huazhong University of Science and Technology (NO.2015S508).

### Preparation of BBR and Treatment Methods

The treatment groups included BBR treatment groups with BBR at high (BH group), middle (BM group), or low dose (BL group) and metformin group (ME group). BBR was purchased from Yabang Pharmaceutical Co. Ltd. in China (H20053180) and dissolved with 0.5% carboxymethyl cellulose sodium glue. High, medium and low dosages in BBR treatment groups were 375, 187.5 and 93.75 mg⋅kg^-1^⋅day^-1^ respectively (Leng et al., 2004). Daily dose of metformin was 184 mg/kg for the rats in ME treatment group. Daily lavage volume of each rat was 1 ml/100 g weight, and the solution concentration was calculated according to the lavage capacity. In addition, 0.5% sodium carboxymethyl cellulose solution was intragastric administrated to the rats in NO and MO groups. Gastric irrigation doses were adjusted weekly based on rats’ weights for 9 weeks.

### Sampling and Metabolic Studies

When the interventions were over, six rats in each group were used for the detections of intestinal permeability and the other six rats in each group were employed for metabolic studies and sampling. When performing OGTT, rats were overnight fasting for 12 h and 2 g/kg glucose was administrated. The blood from tail veins of each rat were collected at 0, 30, 60, and 120 min after the intragastric administration and the blood glucose levels were detected using a glucose monitor (Roche, German).

At the end of 9 weeks’ treatment, all experimental animals were killed. Anesthesia was performed by intraperitoneal injection with 50 mg/kg pentobarbital sodium. Abdominal aorta blood sampling, intestines and MLN were collected under sterile conditions. Samples were fixed with 4% formaldehyde or preserved in -80°C.

The concentration of serum insulin was detected by using iodine radioimmunoassay kit (Jinding, China). For the detection of triglyceride (TG) content, colorimetric assay (Mingdian, China) was used.

### Intestinal Histological Morphology Studies

Intestine sections were stained using hematoxylin and eosin (H&E) to assess the histological morphology. Transmission electron microscope (Hitachi, Japan) was used to evaluate the ultrastructural changes. Tissue blocks were fixed with glutaraldehyde. After rinsing and dehydration in ethanol, the tissues were embedded and sliced with ultramicrotome. Following uranyl acetate and lead citrate double staining, transmission electron microscope was used for the morphology observation and taking pictures.

### Intestinal Permeability Test

FITC-dextran (Sigma, FD-4, USA) perfusion assay was used to detect intestinal epithelial permeability in the rats. After anesthesia, abdominal middle incision and ileum incision 5 cm distance from the appendix was performed and 10-cm-long ileal segment was ligated with 2.0 silk thread. Subsequently, 1ml FITC-dextran (10 mg/ml) was affused into the ligated intestinal cavity. Intestines were returned back to the abdominal cavity and kept wet by wrapping moist gauze and a plastic film. Thirty minutes later, blood was collected from the abdominal aorta. Serum was separated and fluorescence was detected with a spectrophotometer (Synergy2, USA).

### Flow Cytometry

Mesenteric lymph nodes was grinded with strainer mesh and glass rods in the 1640 culture medium containing 2% FBS. Erythrocyte was lysed and the supernatant was dismissed after centrifugation. If the markers were expressed in the cytoplasm and nucleus, FIX/PERM liquid was added to break the cell membrane. Otherwise, the cells were incubated with different antibodies for 30 min away from light and fixed with 1% paraformaldehyde. T cells were stained with CD3-FITC, CD4-APC and CD8-PE (BD Bioscience, USA); Macrophages were with CD11b-APC (BD Bioscience, USA) and CD68-FITC (AbD Serotec, UK), Tregs were with CD4-APC and Foxp3-PE (eBioscience, USA); dendritic cells (DC) were with OX62-FITC (Abcam, UK). Flow cytometry (BD FACSCalibur, USA) was applied for the detection of cells’ population.

### Immunofluorescence Test

Intestinal tissues were separated, rinsed, fixed and cut following paraffin embedding. After using the spontaneous fluorescence quenching agent, the sections were immunostained overnight with primary antibodies against NF-κB (CST, USA) with triton damaging cell membranes. Then fluorescence second antibodies were co-incubated at room temperature for 30 min. Following the rinse, the sections were stained with DAPI (Sigma, USA) for 3 min and sealed. Finally, observation and taking photos were performed under a Nikon fluorescence microscope.

### Real-Time PCR Assay

After the extract of intestinal RNA with Trizol reagent (Takara, China) and concentration determination of RNA, reverse transcriptase kit (Takara, China) was used to synthetize the cDNA. Then, amplification was conducted using SYBR premix EX Taq^TM^ kit (Takara, China) in StepOne PCR detetor (Stepone, USA). Relative expressions of mRNA were calculated by 2^-ΔΔCT^. The primer sequences are listed in **Table 1**.

**Table 1:** Primers for RT-PCR assay.

Gene	Forward (5′→3′)	Reverse (5′→3′)
LBP	CCCAGACGCTGGATGTGATG	TGATCTGAGATGGCAAAGTAGACC
CD14	CTTATGCTCGGCTTGTTGCTGT	TAGCAGCGGACACTTTCCTCGT
IL-1β	GTCGGGACATAGTTGACTTCAC	GACTTGGCAGAGGACTTCAC
TNF-α	CCAGGTTCTCTTCAAGGGACAA	GGTATGAAATGGCAAATCGGCT
IL-10	GCTGGACAACATACTGCTGAC	AATGCTCCTTGATTTCTGGG
IL-4	CTGTCACCCTGTTCTGCTTTCTC	TTTCTGTGACCTGGTTCAAAGTGT
TGF-β	AAGGACCTGGGTTGGAAGT	CGGGTTGTGTTGGTTGTAGA
IL-6	AATCTGCTCTGGTCTTCTGGA	CAGTATTGCTCTGAATGACTCTGG
MIF	GACTTTTAGTGGCACGAGCG	GCTTGCTGTAGTTGCGGTTCT
β-actin	AGCCATGTACGTAGCCATCC	CTCTCAGCTGTGGTGGTGAA

### Western Blot Assay

Proteins of intestines (different sampling volumes for various antibodies) were separated through gel electrophoresis, transferred onto NC membranes, and incubated with antibodies against TLR4 (NOVUS, USA), Occludin-1 (OCLN, Gene Tex, USA), ZO-1 (Invitrogen, USA), Claudin-1 (Abcam, UK), MyD88 (CST, USA), p-IKKβ (Gene Tex, USA),IKKβ (Gene Tex, USA) and β-actin (Affinity, USA). The membranes were then co-incubated with fluorescent second antibodies. Oddesy imaging system was implemented to analyze the protein expressions.

### ELISA

Amylin, GLP-1, GIP and Ghrelin (Ray Biotech, USA) levels in serum were tested using ELISA Kit in line with the competitive enzyme immunoassay principle. In brief, 100 μL serum was added to each well and OD values were detected at 450 nm using a spectrophotometer (Synergy2, USA).

### Statistical Analysis

Data were analyzed by SPSS 20.0 software. Statistical significance was assessed by ANOVA following K-S normality test. With homogeneity of variance, significance between different groups was evaluated by Bonferroni test; alternatively, Dunnett’s T3 test was used. *P*-values < 0.05 suggested that significant differences existed.

## Results

### BBR Attenuates the Disturbance of Glucose and Lipid Metabolism in Diabetic Rats

**FIGURE 1:**
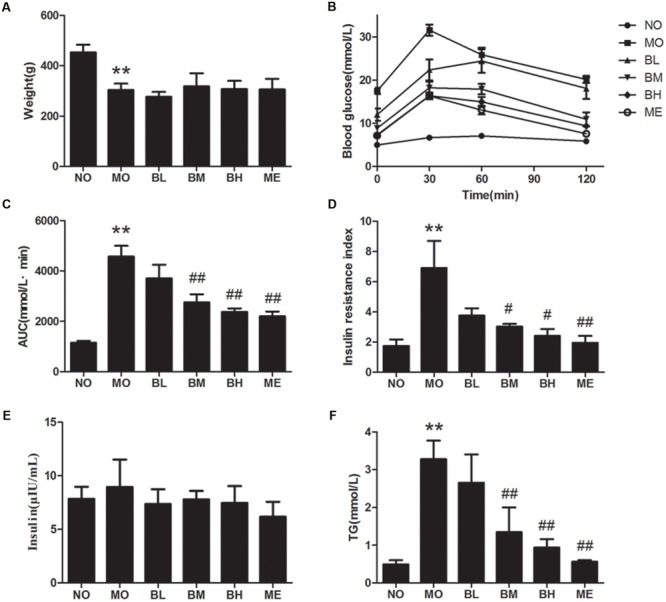
**Berberine (BBR) is effective on maintaining glucose homeostasis. (A)** Body weights of rats in each group. **(B)** OGTT of the rats after an oral gavage of 2.0 g/kg glucose. Data presented as mean ± SE. *P* < 0.01 for NO vs. MO group at each time point; *P* < 0.05 for MO vs. BM group, MO vs. BH group and MO vs. ME group at each time point; *P* < 0.05 for MO vs. BL group at 30 min after glucose administration. **(C)** AUC in OGTT. **(D)** IRI after the treatment. **(E)** Serum insulin concentration of the rats. **(F)** Serum TG concentration. NO, normal control group; MO, diabetic model group; BL, BBR treatment group with a low concentration; BM, BBR treatment group with a middle concentration; BH, BBR treatment group with a high concentration; ME, metformin treatment group; Data presented as mean ± SD except OGTT. *n* = 6 rats in each group. ^∗∗^*P* < 0.01 vs. NO group, ^#^*P* < 0.05 vs. MO group, ^##^*P* < 0.01 vs. MO group.

Firstly, we detected the effects of BBR on glucose and lipid metabolism in diabetic rats (**Figure 1**). Consistent with previous study, diabetic rats underwent the weight loss after modeling (Wang et al., 2016), and BBR interventions did not significantly improve the decreased body weights. With 2 g/kg glucose lavage in OGTT, the blood glucose at each time-point increased significantly in diabetic rats, whereas BBR at middle (187.5 mg/kg) and high dosage (375 mg/kg) reversed elevated fasting and postprandial blood glucose. The area under the concentration-time curve (AUC) in OGTT and serum TG levels also decreased in BBR-treated rats. Although no significant difference was shown with regards to serum insulin concentration in different groups, BBR decreased insulin resistance index (IRI) as well as TG in diabetic rats of BM and BH groups. These data indicated that BBR had specific beneficial effects on metabolic parameters. Similar hypoglycemic and lipid-lowering effects were observed in ME group that metformin was administrated as a positive control.

### BBR Changes the Proportion of Intestinal Immune Cells in MLN of Diabetic Rats

**FIGURE 2:**
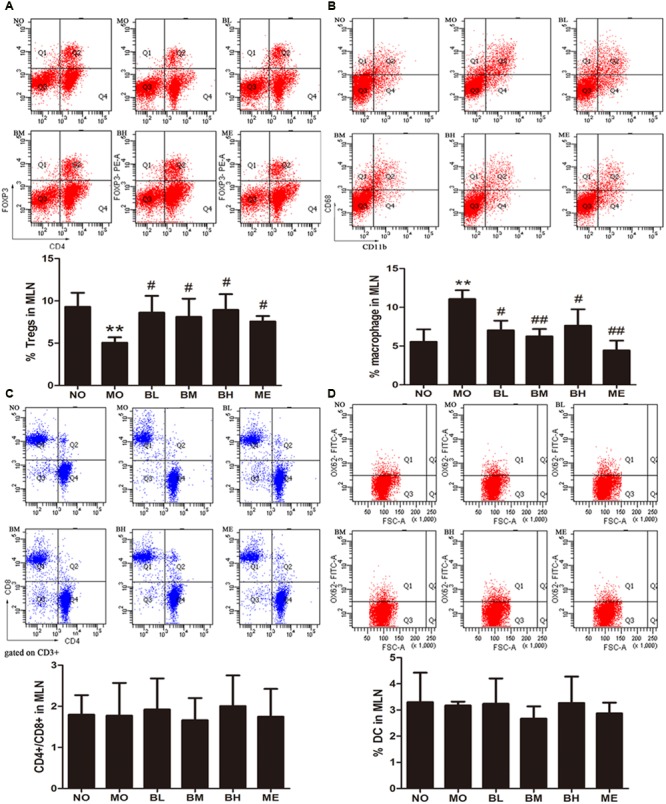
**Berberine increased Tregs and reduced macrophages percentages in MLN of diabetic rats. (A)** Tregs populations in MLN (*n* = 4). **(B)** Macrophage populations of different groups in MLN (*n* = 4 or 5). **(C)** Ratio of CD4+ and CD8+ cells on CD3+ gate (*n* = 5 or 6). **(D)** Dendritic cell populations in MLN (*n* = 3). Data presented as mean ± SD. ^∗∗^*P* < 0.01 vs. NO group, ^#^*P* < 0.05 vs. MO group, ^##^*P* < 0.01 vs. MO group.

Flow cytometry was used to detect the proportions of T cells, macrophages and DC in MLN. As shown in **Figure 2**, the percentage of Tregs in MLN of diabetic rats was decreased while it was increased in BBR- and metformin-treated rats. Because macrophages are the sources of many proinflammatory factors, we studied the proportion differences of macrophages with BBR administration. Significantly higher numbers of CD11b + CD68+ MLN cells were observed in MO group while less numbers were observed in BBR treatment and ME groups. As far as CD4 + T cells, CD8+ T cells, the ratio of CD4+ to CD8+ cells and DC were concerned, no significant changes were found.

### BBR Affects the Expressions of Intestinal Immune Factors in Diabetic Rats

**FIGURE 3:**
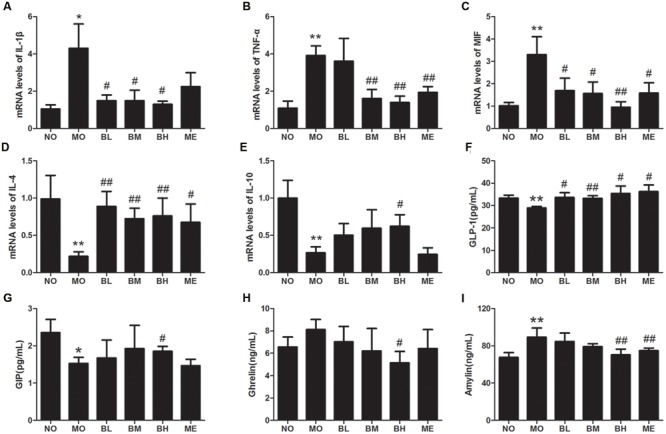
**Berberine affected mRNA expressions of intestinal cytokines and plasma gastrointestinal hormones. (A)** IL-1β mRNA levels. **(B)** TNF-α mRNA levels. **(C)** MIF mRNA levels. **(D)** IL-4 mRNA levels. **(E)** IL-10 mRNA levels. **(F)** Plasma GLP-1 concentrations. **(G)** Plasma GIP concentrations. **(H)** Plasma Ghrelin concentrations. **(I)** Plasma Amylin concentrations. The levels of mRNA were expressed relative to the β-actin. Data presented as mean ± SD. *n* = 6 for each group. *P < 0.05 vs. NO group, **P < 0.01 vs. NO group, ^#^*P* < 0.05 vs. MO group, ^##^*P* < 0.01 vs. MO group.

Intestinal immune factors involved in the maintenance of intestinal immune tolerance and the intestinal barrier integrity. Since BBR had the beneficial effect on immune cells of intestinal immune system, it might also influence the expressions of intestinal immune factors. The results showed that IL-1β, TNF-α and MIF mRNA expressions were elevated in comparison with NO group, while IL-4 and IL-10 mRNA levels were reduced in intestinal tissue of diabetic rats (**Figures 3A–E**). However, BBR, especially at high doses, showed significant inhibitory effects on the expressions of IL-1β, MIF and TNF-α mRNA. Moreover, BBR treatment also exhibited the increase in IL-4 and IL-10 mRNA expressions in intestinal tissue of diabetic rats. Despite increased tendency for IL-6 mRNA expression and decreased trend for TGF-β mRNA expression in MO group, heterogeneity of variance was large and no statistical difference was found in the treatment groups (Data not shown).

### BBR Regulates the Secretion of Gastrointestinal Hormones in Diabetic Rats

Since gastrointestinal hormones participate in glycemic control, we next examined the effects of BBR on plasma gastrointestinal hormones levels (**Figures 3F–I**). After HGFD and the administration of STZ, GLP-1 and GIP secretion reduced while pancreatic beta cells-derived Amylin increased in diabetic rats (**Figures 3F,I**). The administration of BBR at different concentrations increased the secretion of GLP-1, but only BBR at a high dose influenced the production of GIP and Amylin. In the analysis regarding Ghrelin, BBR at a high dose decreased Ghrelin production compared with MO group, and no differences were found between other different groups.

### BBR Improves Intestinal Mucosal Barrier Function in Diabetic Rats

**FIGURE 4:**
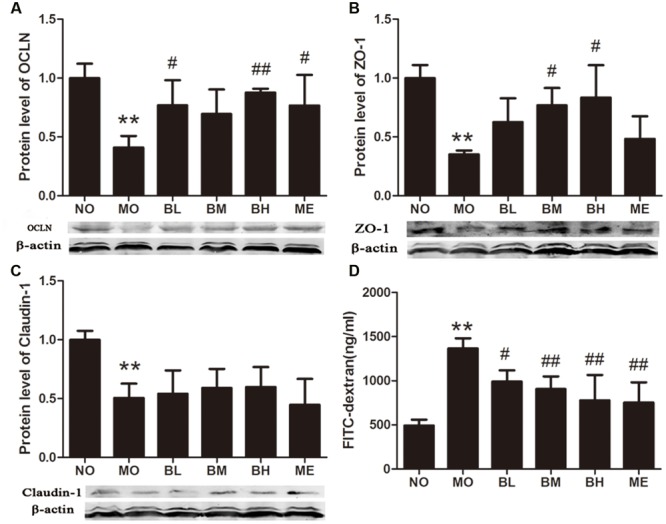
**Berberine protects intestinal mucosal integrity. (A)** OCLN expression in intestine. **(B)** ZO-1 expression in intestine. **(C)** Claudin-1 expression in intestine. **(D)** Serum dextran concentration after the lumina infusion of FITC-dextran. The protein sampling mass was 150 or 200 μg. Data presented as mean ± SD. *n* = 3 for the detections of OCLN, ZO-1 and Claudin-1 expressions; *n* = 6 for serum dextran concentration. ^∗∗^*P* < 0.01 vs. NO group, ^#^*P* < 0.05 vs. MO group, ^##^*P* < 0.01 vs. MO group.

**FIGURE 5:**
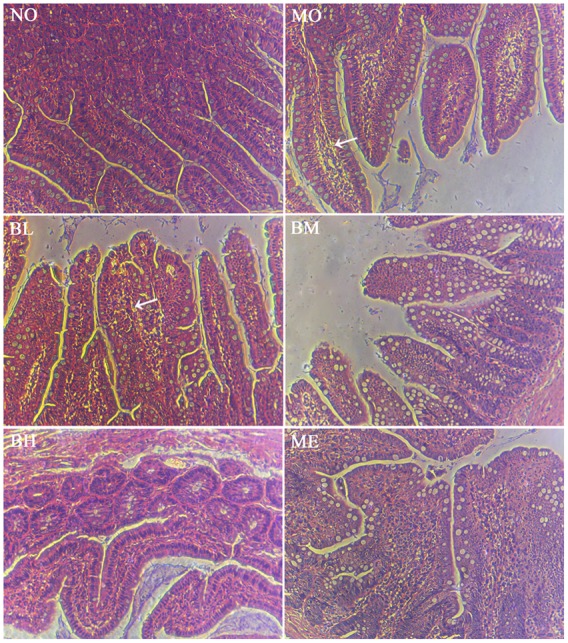
**Histology of small bowel with H&E staining after the intestinal infusion of FITC-dextran.** FITC-dextran was different under the intestinal epithelium after intestinal perfusion. White arrowheads suggested more fluorescent dextran underneath the intestinal epithelium was observed.

**FIGURE 6:**
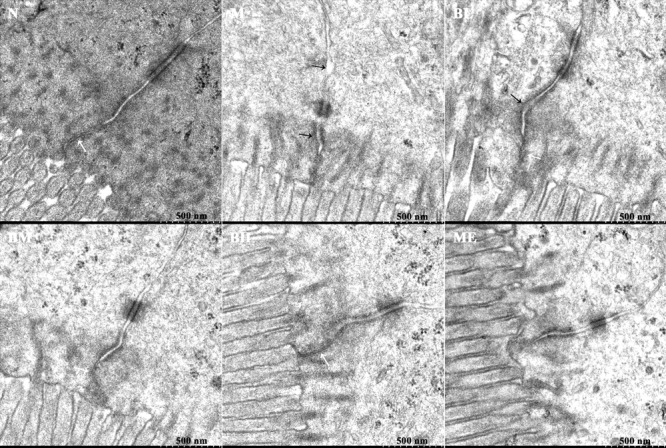
**Ultrastructure of intestinal intercellular junction in different groups.** The figures revealed the disrupted and expanded adherens junctions of intestinal epithelial cells in MO and BL groups, although the desmosome was visible in each group. Black arrowheads indicated damaged cell-cell adhesion and wider gaps between adjacent intestinal epithelial cells. White arrowheads suggested the tight junctions.

High glucose and fat diet and intestinal inflammation could disrupt the intestinal mucosal barriers, leading to endotoxemia and the development of DM. Thus, we studied the effects of BBR on intestinal tight junction proteins expressions and mucosal barrier permeability. Tight junction protein OCLN, ZO-1 and Claudin-1 expressions were detected using western blot. Compared with the rats in NO group, these tight junction proteins expressions were reduced in the intestine of untreated diabetic rats. BBR treatment could partially increase OCLN and ZO-1 expressions but it showed no effects on Claudin-1 levels (**Figures 4A–C**). After the intestinal perfusion of FITC-dextran, BBR could reduce serum dextran concentration, namely the intestinal barrier permeability (**Figure 4D**). Although HE staining illustrated no significant change in intestinal structure between these groups (**Supplementary Figure S1**), the amount and distribution of filtered FITC-dextran were different in intestinal mucosa and submucosa tissues after intestinal dextran perfusion (**Figure 5**), which also suggested differences of the intestinal permeability in the rats of various groups. With regard to the ultrastructure of intestinal epithelial cells, intercellular adhesions were damaged, and the adherent junction in diabetic rats was sparse and expanded. These changes were improved after BBR treatment (**Figure 6**).

### BBR Modulates the Expressions of the Molecules Involved in TLR4/MyD88/NF-κB Signaling Pathways in Intestines of Diabetic Rats

**FIGURE 7:**
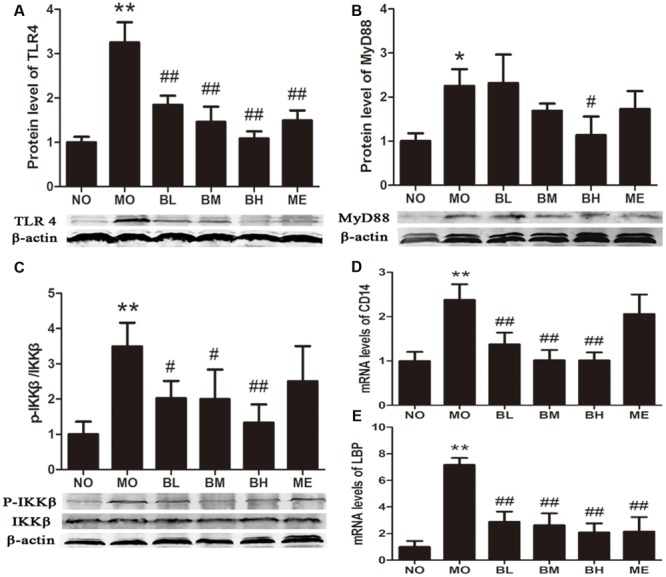
**The gut-protective effects of BBR are related to TLR4/MyD88/NFκB signaling pathway. (A)** TLR4 expressions in intestine (*n* = 4). **(B)** MyD88 expressions in intestine (*n* = 3). **(C)** Fold change of p-IKKβ/IKKβ ratios in intestine (*n* = 4). **(D)** The mRNA levels of CD14 (*n* = 6). **(E)** The mRNA levels of LBP (*n* = 6). The levels of mRNA were expressed relative to the β-actin. The protein sampling mass was 200 μg for TLR4 and MyD88; while 100 μg for p-IKKβ and IKKβ. Data presented as mean ± SD. ^∗^*P* < 0.05 vs. NO group, ^∗∗^*P* < 0.01 vs. NO group, ^#^*P* < 0.05 vs. MO group, ^##^*P* < 0.01 vs. MO group.

**FIGURE 8:**
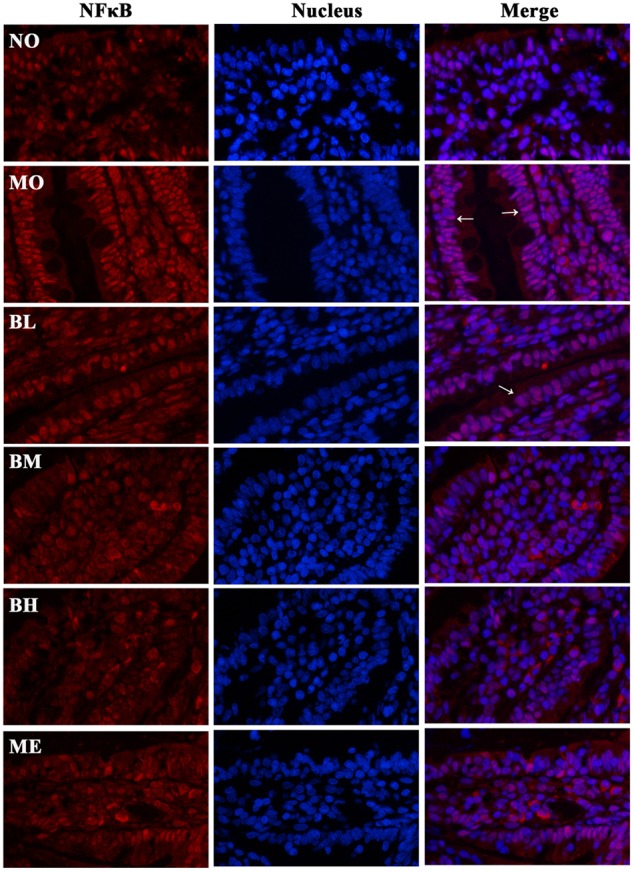
**Representative immunofluorescence staining images of p65 NF-κB in small bowels.** Transcription factor NF-κB is widely expressed in the intestines, and the distribution in nucleus and cytoplasm is different under various conditions. The results suggested the distributions of p65 NF-κB (red) were different in the cytoplasm and nuclei of intestinal epithelium among different groups. Nuclei were stained blue with DAPI. BBR might reduce the translocation of p65 NF-κB into the nuclei (pink). White arrowheads indicated the distributions of p65 NF-κB in the nuclei of intestinal epithelial cells.

Previous studies confirmed that BBR could protect islet beta cells in DM, and also prevent intestinal inflammation in bowel disease through depressing TLR4 signaling pathways (Zhang et al., 2011). To ascertain whether the hypoglycemic and gut-protective effects of BBR were mediated through anti-inflammatory signal transduction pathways, we detected the expressions of TLR4 and downstream MyD88 and p-IKKβ in the intestines, along with p65 NF-κB distributions in epithelial cytoplasm and nucleus. The results revealed BBR, especially at a high dose, reduced intestinal TLR4 and MyD88 protein expressions as well as the phosphorylation of IKKβ (**Figures 7A–C**). As a main ligand for TLR4, LPS combined with LPS binding protein (LBP) and CD14 to form the trimer; then the complex binds to the TLR4 and activates inflammatory signal transduction pathways. Data of RT-PCR illustrated intestinal LBP and CD14 mRNA levels decreased in BBR-treated rats (**Figures 7D,E**). Accordingly, **Figure 8** showed p65 NF-κB distributions were different in intestinal epithelial cells among different groups, and BBR exerted a potential ability to blunt p65 NF-κB translocation to the nuclei.

## Discussion

T2DM rat model was established by HGFD and intravenous injection of 24 mg/kg STZ in this study (Duca et al., 2015). Consistent with previous studies, decreased body weights were observed after the STZ treatment (Wang et al., 2016). After BBR delivery with the dose range between 93.75 and 375 mg/kg, the hypoglycemic and hypolipidemic efficacies were shown in a dose-dependent manner. Previous researches have shown that the bioavailability of BBR was lower than 5% (Pirillo and Catapano, 2015), and it seems inexplicable for the valid hypoglycemic effects with such low bioavailability. We then investigated whether BBR exerted beneficial therapeutic impacts on DM through the intestines.

Though human and rodent studies confirmed the proportion changes of immune cells in gut LP of diabetic mice (Monteiro-Sepulveda et al., 2015), the transplantation of MLN can transmit diabetes to the recipient mice, which highlights the vital pathogenic effects of MLN (Antvorskov et al., 2014; Winer et al., 2016). Therefore we firstly focused on the immune cells population alterations in the MLN of diabetic rats. Consistent with the changes in intestinal LP, we observed reduced Tregs and increased macrophages in diabetic rats, which was inhibited by BBR treatment. Previous studies also found CD4+, CD8+ and DC cells went through transformations in LP (Monteiro-Sepulveda et al., 2015), but no significant differences about aforementioned cells were discovered in the present experiment. It might be explained by different high fat diet or treatment duration, which led to the differentia in intestinal bacteria spectrum and the variant activated immune cells. Based on proinflammatory change of immune cells, we observed that intestinal inflammatory cytokines IL-1β, TNF-α and MIF-1 increased, and IL-10 and IL-4 which maintain immune tolerance reduced in diabetic rats in the present study. These intestinal cytokine changes in diabetic rats were consistent with the data in clinical trials; namely, intestinal cytokine’s milieu of healthy persons features high levels of sIgA and IL-10 (Fritz et al., 2012) while that of subjects with metabolic disease features increased proinflammatory cytokines in the intestinal LP and epithelium (Garidou et al., 2015).

How did these pro-inflammatory changes occur in intestinal immune system of diabetic rats? How did they affect the development of DM? HGFD was found to induce decreased expressions and abnormal distributions of intestinal tight junction proteins, and then intestinal permeability was increased (Everard et al., 2013; Wang et al., 2014). Once the mucosal barrier was damaged, luminal LPS, DNA and other bacterial products went through bowels, activated the immune cells with the expressions of CD14 and TLR4 (Delzenne et al., 2015; Plociennikowska et al., 2015; Winer et al., 2016), and caused abnormal secretions of immune cytokine (He et al., 2016). With proinflammatory cytokines binding to responding receptors, insulin signaling pathways were interfered, and abnormal insulin secretion and insulin resistance emerged (Caricilli et al., 2011). Besides, in chronic inflammatory state of DM, proinflammatory cytokines such as IL-1β, IL-6 and TNF-α aggravated the impairment in intestinal mucosal barrier (Winer et al., 2016). In the present study, tight junction proteins OCLN, ZO-1 and Claudin-1 expressions were decreased, and serum FITC-dextran was increased after the luminal dextran perfusion in diabetic rats. BBR seemed to protect the intestinal barrier integrity through modulating ZO-1 and OCLN expressions; however, BBR did not influence the Claudin-1 level in our study. Claudin-1 is a member of claudin family and an essential component in intestinal mucosal barrier forming. The intestinal proinflammatory immune factor and NF-κB could induce the decrease in claudin-1 expression inflammatory bowel disease (Zhou et al., 2009), and BBR might regulate claudin-4 and claudin-2 expressions other than claudin-1 in claudin family (Amasheh et al., 2010; Li et al., 2014). In the present study, we also found that BBR could blunt TLR4 expression and attenuate local metabolic inflammation possibly through suppressing TLR4/MyD88/IKKβ signaling pathways in intestinal tissue. It might be one of the molecular mechanisms for BBR to protect the intestinal mucosal barrier and inhibit the proinflammatory changes in the treatment of DM.

Additionally, we observed the secretion of GLP-1 and GIP was reduced in diabetic rats, and the concentration of Amylin was increased despite of no significant change of Ghrelin. BBR, especially at a high concentration, prevented the aberrant changes of the hormones, which might be related to its inhibitory effects on intestinal proinflammatory cells and cytokines. These gastrointestinal hormones GLP-1, GIP and Ghrelin, and pancreatic beta cells-derived Amylin, can modulate glucose metabolism (Westwell-Roper et al., 2014). Intestinal immune molecules could also affect GLP-1 secretion from ileal L cells, and anti-TNF-α therapy could reverse high fat diet induced reduction of GLP-1 in diabetic mice (Everard et al., 2013; Anhe et al., 2015; Luck et al., 2015; Yusta et al., 2015). Doubtfully, Ghrelin concentrations in the rats of NO group appeared to be higher than those of BH group despite of no statistically significance. Ghrelin was secreted from gastric P/D1 cells and pancreatic 𝜀 cells, which was influenced by many factors such as STZ, HGFD, gut microbiota, bitter taste receptors, and so on (Janssen et al., 2011); STZ could increase its secretion (Muller et al., 2015) while high-fat diet could reduce its secretion (Little and Feinle-Bisset, 2011). Previous study had proved that BBR might regulate the secretion of GLP-1 via intestinal bitter taste receptor in enteric L cells (Yu et al., 2015). Further studies are required to confirm whether BBR could influence bitter taste receptors in ghrelin-secreting cells.

The intestinal improvements in the diabetic rats receiving BBR administration gave rise to question whether intestines were the exclusive hypoglycemic action sites of BBR. When the absorption rate of BBR was enhanced, the hypoglycemic effect was better (Zhaojie et al., 2014; Pirillo and Catapano, 2015). Therefore, BBR may exert its anti-diabetic effect through multiple ways, and the target in the intestine is probably just one of them. Consistent with preceding studies, the positive control metformin could promote the secretion of GLP-1 (McCreight et al., 2016). Although metformin had obvious gastrointestinal side-effect in patients, it exhibited a protective effect on the expression of OCLN. In addition, metformin could reduce TLR4 expressions, increase Tregs and decrease the proportion of macrophages. Such effects might be related to the increased expression of intestinal probiotics as previously reported (Forslund et al., 2015).

Intestinal immune system, endocrine system, mucosal barrier and gut microbiota interact with each other and have cross-talks, but the initial lesion and subsequent reaction are not fully clear. Further elaborate *in vivo* and *ex vivo* experiments need to be performed to confirm BBR preliminary acts on one or some of the sites. Taken together, these findings suggested the abnormities of intestinal mucosal barrier, immune system, endocrine and PPRs in diabetic rats. However, BBR could improve the barrier function defect, depress the proinflammatory changes of intestinal immune cells and cytokines, and change aberrant gut-derived hormones which might be pharmacological mechanisms for treating diabetes. However, the interaction among them and the exact anti-diabetic mechanism of BBR need to be further studied.

## Author Contributions

FL, HD, and JG designed the study. JG, MH, ZH, KF, QC, JL, DY, and XZ conducted the experiments. JG, HD, KF, and FL wrote and revised the manuscript. DW, LX, and KW also contributed to the design. All authors approved the final version to be published.

## Conflict of Interest Statement

The authors declare that the research was conducted in the absence of any commercial or financial relationships that could be construed as a potential conflict of interest.

## Supplementary Material

FIGURE S1 **Histology of small bowel with H&E staining in each group.** No obvious pathological differences were shown in intestinal tissue histology with H&E staining among NO, MO and various treatment groups.
