# Supplementary figures and images for: Berberine Attenuates Intestinal Mucosal Barrier Dysfunction in Type 2 Diabetic Rats
Source: Front Pharmacol. 2017 Feb 3;8:42. doi: 10.3389/fphar.2017.00042 (PMC5290458; doi:10.3389/fphar.2017.00042)

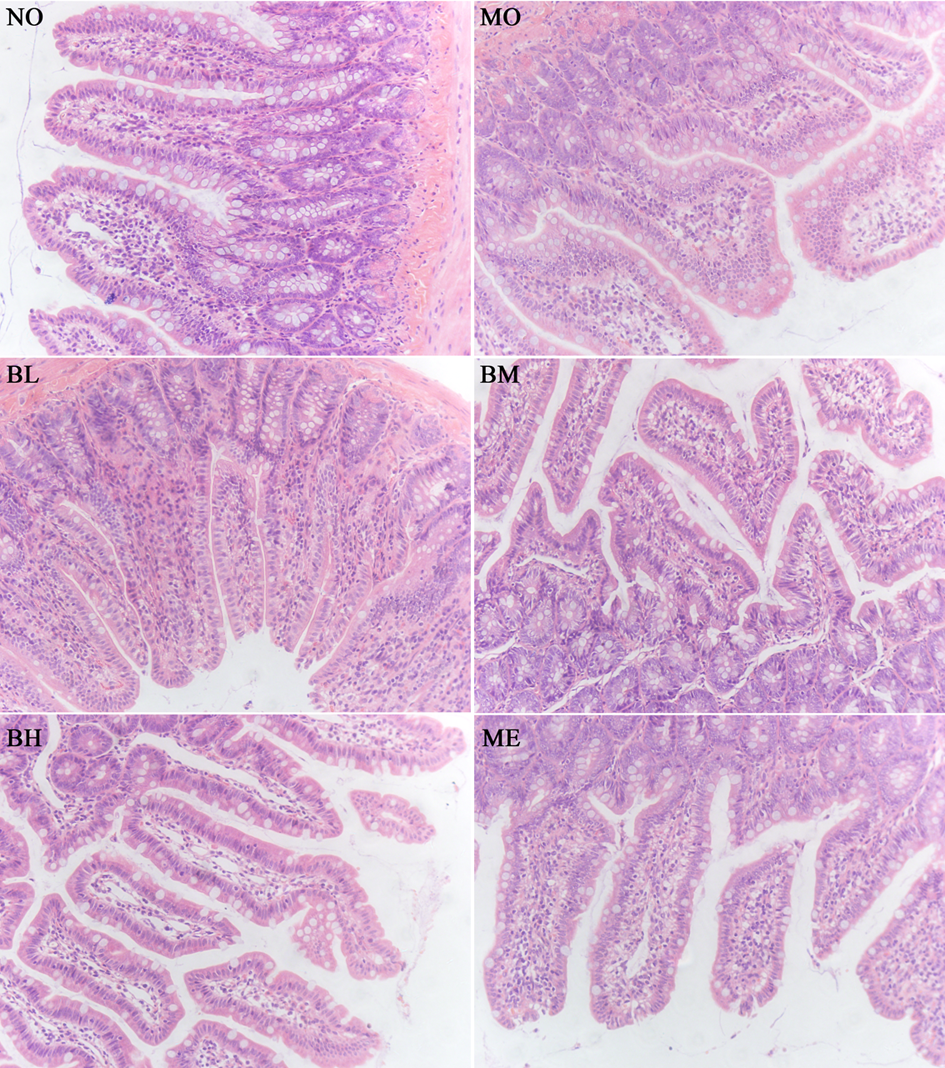

Supplement: FIGURE S1 — Histology of small bowel with H&E staining in each group. No obvious pathological differences were shown in intestinal tissue histology with H&E staining among NO, MO and various treatment groups. [file Image_1.TIF]
